# Intersigmoid Hernia: A Forgotten Diagnosis—A Systematic Review of the Literature over Anatomical, Diagnostic, Surgical, and Medicolegal Aspects

**DOI:** 10.1155/2020/4891796

**Published:** 2020-06-01

**Authors:** Stella Chiarini, Paolo Ruscelli, Roberto Cirocchi, Vito D'Andrea, Beatrice Sensi, Alberto Santoro, Alessia Corsi, Federico Zepponi, Piergiorgio Fedeli, Sara Gioia

**Affiliations:** ^1^Department of Medicine and Ageing Science, University of Chieti, Via Dei Vestini 31, Chieti, Italy; ^2^Emergency Surgery Unit, Torrette Hospital, Polytechnic University of Marche, Via Conca 71, Torrette 60020, Ancona, Italy; ^3^Department of Surgical Sciences, University of Perugia, Piazza Dell' Università 1, Perugia 06100, Italy; ^4^Department of Surgical Sciences, Sapienza University of Rome, Piazzale Aldo Moro 5, Rome 00185, Italy; ^5^Law School, University of Camerino, Via A. D'Accorso 16, Camerino, MC, Italy

## Abstract

**Introduction:**

Intersigmoid hernia is a hernia of the small bowel into the intersigmoid fossa. It is well known to be a rare condition. Recent reports reveal that the preoperative differentiation of intersigmoid hernias is difficult and the diagnosis is often confirmed during the laparotomic exploration. Due to the vague clinical manifestation in most cases, the surgical treatment is frequently delayed.

**Materials and Methods:**

In this study, we systematically reviewed the literature up to 2019 covering 114 studies and 124 patients with an intersigmoid hernia. The purpose of this work is to improve the understanding of the anatomical aspects, clinical presentation, diagnosis, and treatment of intersigmoid hernia so as to assist the preoperative differentiation of these hernias when presented as acute abdomen in the emergency department.

**Results:**

The diameter of the intersigmoid recess was reported with mean 2.65 cm (range 1–10 cm, SD 1.15 cm) and the length of the incarcerated small intestine was between 3 cm (min) and 150 cm (max): mean 25.25 cm, SD 35.04 cm. The diameter of the sigmoid recess was greater in patients who underwent resection due to strangulation (mean 3.31 cm, SD 1.53 cm) compared to those who underwent only reduction of the hernia (mean 2.35 cm, SD 0.74 cm). The time from onset to operation was less in patients undergoing resection surgery due to throttling (mean 3.03 days, SD 3.01 days) compared to those who underwent only a reduction of hernia incarceration (mean 8.49 days, SD 6.83 days).

**Conclusion:**

Intersigmoid hernia is often a forgotten diagnosis and a clinical challange due to its anatomical characteristics.

## 1. Introduction

**Figure 1:**
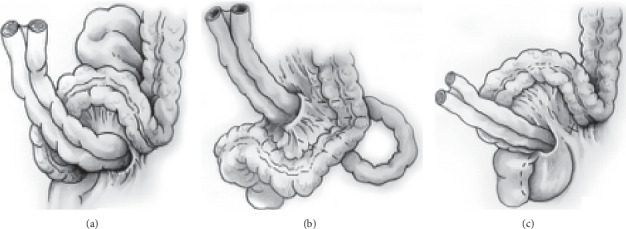
Sigmoid mesocolon hernia is classified into 3 types: (a) intersigmoid hernia, (b) transmesosigmoid hernia, and (c) intramesosigmoid hernia.

Internal hernias account for less than 6% of all cases with small bowel ileus and are considered by some authors as “the forgotten differential diagnosis” [1]. Benson and Killen classified sigmoid mesocolon hernia into 3 types (Figure 1): (1) intersigmoid hernia that involves herniation into the sigmoid fossa (the most common), (2) transmesosigmoid hernia: passing of intestinal loops through an isolated defect in the sigmoid mesocolon with no hernia sac, and (3) intramesosigmoid hernia: herniation through a congenital defect in the lateral peritoneal surface of the mesocolon not related to the sigmoid fossa [2].

**Figure 2:**
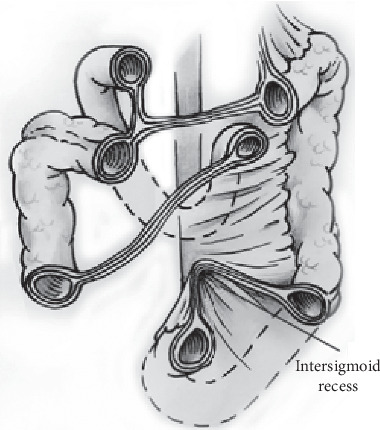
Intersigmoid hernia (ISH).

Intersigmoid hernia (ISH) is defined as the herniation of the small intestine into the intersigmoid fossa (ISF), which is a congenital V-shaped recess of the peritoneum situated at the lateral attachment of the sigmoid mesocolon (Figure 2). This space is very small and in consequence the herniation loop is very short [3]. Furthermore, patients with ISH have few specific clinical signs, and therefore, it is often diagnosed after an explorative laparotomy. ISH should be suspected in those cases of intestinal obstruction with no surgical history [4]. Recently, laparoscopic surgery has been broadly performed for small bowel obstruction. Laparoscopic surgery has not only high diagnostic value but also minimal invasiveness in comparison with open surgery even in emergency surgery [5, 6]. Therefore, because ISH has few specific clinical and radiological findings, laparoscopic surgery has clear advantages [7, 8].

We systematically reviewed the literature from 1964 to 2019 covering 114 studies and 124 patients with ISH. The scope of this work is focused on the anatomical aspects, clinical presentation, diagnosis, and treatment in order to improve the preoperative differentiation of these hernias and their surgical management.

## 2. Method

We systematically reviewed the literature to evaluate the data related to the intersigmoid fossa using the “The Standard for Reporting Items for Systematic Review and Meta-Analysis” (PRISMA). One search was conducted using PubMed, SCOPUS, and the Web of Science databases on 10 April 2019 using the following keywords: intersigmoid [All Fields] AND fossa [All Fields]; intersigmoid [All Fields] AND (“hernia”[MeSH Terms] OR “hernia”[All Fields]) with no language restrictions. All the data were reviewed by Vito D'Andrea and Alessia Corsi. In cases where an article had been published by the same group and there was an overlap of the research period, only the latest study was included in order to avoid duplication of data. The PubMed “related articles” option was used to extend the search, and the list of references for the studies found was examined. In addition, a search of the grey literature was performed on Google Books (https://books.google.com).

Vito D'Andrea and Alberto Santoro developed a data mining technique based on the “Cochrane Consumers and Communication Review Group” model and independently extracted data from the selected studies. The following information was extracted from each study: the name of the principal author and the year of publication, the country where the study was performed, the type of the study, and the number of patients enrolled.

Outcomes were the evaluation of the following characteristics:

- The anatomic characteristics of the intersigmoid recess
- The characteristics of the herniated bowel
- The characteristics of surgical approach

## 3. Results

The present systematic review of the literature comprised 114 studies (SDC 1) in which 124 cases of ISH were reported (SDC 2). Most of the studies were carried out in Asia (102 studies). Only a few studies were performed in Europe (8 studies), North America (3 studies), and Africa (1 study). All patients arrived at the emergency department with a diagnosis of intestinal obstruction. In 21 cases, previous abdominal or pelvic surgery had been performed (appendectomy in 33%). For 124 patients, their ages had a mean of 55.5 years with a standard deviation (SD) of 16.36. The diagnostic modalities were reported in 120 cases (SDC 3). In 104 cases, a plain abdominal X-ray was performed and/or an abdominal CT scan in 87 patients. In the majority of patients (74%), a step-up approach was chosen. In 96 patients, the diameter of the intersigmoid recess was reported with mean 2.65 cm (range 1–10 cm, SD 1.15 cm). The length of the incarcerated small intestine was reported in 70 patients as 3 cm (min) and 150 cm (max): mean 25.25 cm, SD 35.04 cm. In 2 cases, the treatment was not described [9, 10]. In the other 122 patients, for 86 patients, only the reduction of the incarceration was performed (70.5%), and for the remaining 36 cases, additionally, the resection of the strangulated loop due to necrosis was necessary (29.5%) (SDC 4). The diameter of the sigmoid recess was greater in patients who underwent resection due to strangulation (mean 3.31 cm, SD 1.53 cm) compared to those who underwent only reduction of the hernia (mean 2.35 cm, SD 0.74 cm) (SDC 5). The time from onset to operation was less in patients undergoing resection surgery due to throttling (mean 3.03 days, SD 3.01 days) compared to those who underwent only a reduction of hernia incarceration (mean 8.49 days, SD 6.83 days). From 1990 till now, surgery was performed on 106 patients: 75 patients had laparotomy, and 29 of these had a resection, 33 had a laparoscopic reduction of the hernia (4 underwent a laparoscopy with subsequent conversion to a laparotomy with subsequent bowel resection), and 3 patients had a complete laparoscopic resection of the small bowel.

## 4. Discussion

Internal hernias are a protrusion of an internal organ through an anatomical or pathological opening within the limits of the peritoneal cavity. These include paraduodenal, pericaecal, through the foramen of Winslow, transmesenteric, transomental, sigmoid mesocolon, and retroanastomotic hernias.

The ISF, when it exists, is a V-shaped cul-de-sac peritoneal recess with variable dimension and situated between the two roots of the parietal brim of the sigmoid mesocolon (Figure 2). During the process of organ formation in foetal development, after the descending colon has taken up its position on the left side of the peritoneum; the left leaf of the mesentery fuses with the primary parietal peritoneum with the mesentery disappearing and becoming fixed to the retroperitoneum. This usually happens at around 5 months of gestation, but the process of fusion may be delayed or incomplete in the left paracolic gutter region thus forming the ISF. It is relatively common, occurring in 50–75% of autopsy cases. Nevertheless, ISHs are rare and occur in approximately 6% of all internal cases [11]. In fact, we found only 124 patients reported for the period from 1964 to 2019.

All the patients arrived at the emergency ward with intestinal obstruction and 21 had previous abdominal or pelvic surgery (appendectomy 33%). The diagnostic modalities were reported in 120 cases. In 104 patients, an abdominal radiography was performed, along with a CT scan of the abdomen in 87 cases. In most cases, a step-up approach was performed. Contrast enhancement via an nasogastric tube and abdominal contrast enhancement CT were useful tests for the diagnosis of ISH. Nowadays, the contrast abdominal CT is increasingly becoming a gold standard diagnostic tool of the small bowel ileus and internal hernias in particular [12]. Additionally, it may facilitate the planning of the surgical intervention, especially when a laparoscopic approach is attempted. Typical CT findings include intestinal dilatation of the lateral dorsal side of the sigmoid colon, mesenteric accumulation towards the middle line, arc-shaped extension of the sigmoid colon, and folding and twisting of the incarcerated small intestine into a loop or figure of eight [13]. The mean length of the incarcerated small intestine was reported in 70 patients and was 25.5 cm. The mean diameter of the intersigmoid recess was greater in patients undergoing resection due to strangulation and necrosis compared to those who underwent only a surgical reduction of the hernia (3.3 cm vs. 2.3 cm) [14]. The time from onset to operation was shorter in patients who underwent resectional surgery compared to the cases with reduction of hernia incarceration (3 vs. 8.5 days). This delay could be attributed to the vague clinical manifestation of the internal hernias. This is especially true in the cases with spontaneous reduction which leads to fluctuation of the symptoms. The internal hernias can be one of the causes of nonspecific abdominal pain, as frequently addressed by the physicians in the emergency departments [15, 16].

Surgery is the therapeutic strategy for ISH. According to our review, since 1990 to the present day, surgery has been performed on 106 patients with ISH. Seventy-five patients underwent laparotomy, 29 of which were combined with a bowel resection. Thirty-three patients had a laparoscopic reduction of the incarcerated hernia and four of which (12%) underwent subsequent conversion to laparotomy for resection of the small bowel. Finally, three patients had a complete laparoscopic resection of the small bowel. Actually, this conversion rate is acceptable, especially in an emergency setting, but requires careful judgment and advanced laparoscopic skills [6].

Malpractice claims are increasingly important aspects of surgical practice. The Institute of Medicine (IOM) report estimated the total global cost of medical error as USD 17–29 billion per annum [17]. The role of legal medicine has become essential in the judicial setting, especially in the nations with a “fault system” in order to prevent and avoid erroneous interpretations and hasty scientific verdicts [18, 19].

Intersigmoid hernia can become a medicolegal dilemma due to physicians failing to recognize it because of its rarity in clinical practice. In medical malpractice cases, the defendant's behaviour is compared to the standard of care for that specific situation [20]. The standard of care is most commonly defined as “that reasonable and ordinary care, skill, and diligence as physicians and surgeons in good standing in the same neighbourhood, the same general line of practice, ordinarily have been exercising in like cases.”

Regarding ISH, the highest risk is to delay the diagnosis due to the relatively nonspecific clinical presentation. Due to a delayed diagnosis, surgery will also be delayed, with the risk of necessity of conversion to laparotomy.

Therefore, in order to avoid malpractice claims, the standard care for ISH is a stepwise approach, preforming first an abdominal CT scan that represents a valuable diagnostic tool allowing precise localization of the obstruction.

Moreover, a thorough knowledge of the anatomy of the peritoneal cavity and a comprehension of the clinical and radiological findings are mandatory for a correct surgical approach.

## 5. Conclusion

Clinical reports revealed that preoperative differentiation of intersigmoid hernias is difficult, and the diagnosis is often confirmed during the surgical management. Due to the vague clinical manifestation in most cases, the surgical treatment is frequently delayed. Therefore, also to avoid malpractice claims, a stepwise approach is recommended. Nowadays, the abdominal CT scan represents a valuable diagnostic tool allowing precise localization of the abdominal obstruction. An abnormal finding is the diameter of the sigmoid recess that is greater in the cases with strangulation and resection. Most cases can be managed only by reduction of the hernia, while the resection is necessary in one third of the cases. Laparoscopy is a feasible approach but requires advanced skills.

In conclusion, a thorough knowledge of the anatomy of the peritoneal cavity and a comprehension of the clinical and radiological findings will allow a better surgical approach for internal hernias in most cases of acute abdomen.

ISH is a very rare condition and often is a forgotten diagnosis and a challenge due to its anatomical characteristics.

## Supplementary Materials

Supplementary Materials Supplementary digital contents: SDC 1, Table 1: references of included studies, SDC 2, Table 2: characteristics of included studies, SDC 3, Table 3: radiological evaluation of included studies, SDC 4, Table 4: intraoperative findings, SDC 5: type of surgical treatment, and SDC 6: characteristics of the defect and the incarcerated small bowel.
