# Supplementary material for: Intersigmoid Hernia: A Forgotten Diagnosis—A Systematic Review of the Literature over Anatomical, Diagnostic, Surgical, and Medicolegal Aspects
Source: Emerg Med Int. 2020 Jun 1;2020:4891796. doi: 10.1155/2020/4891796 (PMC7285387; doi:10.1155/2020/4891796)
Supplement: Supplementary Materials — Supplementary digital contents: SDC 1, Table 1: references of included studies, SDC 2, Table 2: characteristics of included studies, SDC 3, Table 3: radiological evaluation of included studies, SDC 4, Table 4: intraoperative findings, SDC 5: type of surgical treatment, and SDC 6: characteristics of the defect and the incarcerated small bowel. [file 4891796.f1.pdf]

Supplementary Digital Contents

SDC 1. Table 1: References of included studies

SDC 1: References of included studies

1. Agarwal A, Ray U, Hossain MZ, Mitra N, Das A, Agarwal A, Gupta M. “Congenital intra-mesosigmoid hernia- a case report of a rare sigmoid mesocolon hernia”.Indian J Surg. 2011 Dec;73(6):450-2. doi: 10.1 007/s12262-011-0275-9. Epub 2011 Apr 16. PubMed PMID: 23204707; PubMed Central PMCID: PMC3236271.

2. Agatsuma Y, Iizuka A, Sato B *et al*. “A Case of Strangulated Ileus Caused by Intramesosigmoid Hernia with Left Paraduodenal Hernia”. Nihon Rinsho Geka Gakkai Zasshi (Journal of Japan Surgical Association). 2017 Volume 78 Issue 11 2465-2468 DOI: https://doi.org/10.3919/jjsa.78.2465

3. Ahmad N, Faiz N, Kumari R, et al. “Intramesosigmoid hernia presenting as intestinal obstruction”. J. Evolution Med. Dent. Sci. 2017;6(47):3673-3675, DOI: 10.14260/Jemds/2017/793

4. Akiyama Y, Aoki K, Nakaya T, Fujiwara H *et al*. “Intestinal Obstruction due to an Intramesosigmoid Hernia”. The Japanese Journal of Gastroenterological Surgery. 2004 Volume 37 Issue 11 1781-1786 DOI: https://doi.org/10.5833/jjgs.37.1781

5. Ankit Shukla, Ramesh Bharti, Satish Kumar, Rajesh Chaudhary (Semptember2015). Dealing with intramesosigmoid hernia: A rare variety of internal hernia. Archives of clinical and experimental surgery. DOI: 10.5455/aces20151020060526

6. Bandawar MS, Nayak P, Shaikh IA, Sakthivel MS, Yadav TD. “Strangulated small bowel obstruction secondary to a transmesosigmoid hernia”. Indian J Surg. 2014 Apr;76(2): 148-9. doi: 10.1007/s12262-012-0649-7. Epub 2012 Jul 4. PubMed PMID: 24891782; PubMed Central PMCID: PMC4039682.

7. Bao SQ. “Two cases of mesentery hernia causing strangulation obstruction”. Qiqihaer Yixue Xuebao 2009; 30: 1951-1952

8. Benson JR, Killen DA. “Internal hernias involving the sigmoid mesocolon”. Ann Surg. 1964 Mar;159:382-4. PubMed PMID: 14129383; PubMed Central PMCID: PMC1408577.

9. Benyamini P, Lopez S, Cooper M, Mohamad O, Maldini G. “Congenital Mesenteric Defect: An Uncommon Cause of Bowel Obstruction”. Hawaii J Med Public Health. 2016 Feb;75(2):46-7. PubMed PMID: 26918208; PubMed Central PMCID: PMC4755067.

10. Bircher, Martin Derek, and Anthony Edward Stuart. "Internal herniation involving the sigmoid mesocolon." Diseases of the Colon & Rectum 24.5 (1981): 404-406. PubMed PMID: 7261828.

11. Campbell, Aideen, K. Atkin, Gary. (2015). “Laparoscopic Repair of Incarcerated Intersigmoid Hernia”. CRSLS: MIS Case Reports from SLS. 19. DOI: 10.4293/CRSLS.2013.00214

12. Chou CK. Internal hernia of the small bowel through the sigmoid mesocolon. Int J Radiol Radiat Ther. 2017;2(6):142–144. DOI: 10.15406/ijrrt.2017.02.00044

13. Collins D, Kavanagh D, Myers E, Richards S, McDermott E. "Incarcerated transmesosigmoid hernia presenting in a 60-year-old man: a case report". J Med Case Rep. 2008 May 17;2:161. doi: 10.1186/1752-1947-2-161. PubMed PMID: 18485232; PubMed Central PMCID: PMC2396178.

14. Endo Y, Yoshida T et al. "A case of transmesosigmoid hernia". The Japanese Journal of Gastroenterological Surgery. 2001 Volume 34 Issue 5 510-514 DOI: https://doi.org/10.5833/jjgs.34.510

15. Farah RH, Fahmi Y, Khaiz D, Elhattabi K, Bensardi F, Lefriyekh R, Berrada S, Fadil A, Ouariti NZ. "Post-operative transmesosigmoid hernia causing small bowel obstruction: a case report." Pan Afr Med J. 2015 Apr 1;20:318. doi: 10.11604/pamj.2015.20.318.5752. eCollection 2015. PubMed PMID: 26175809; PubMed Central PMCID: PMC4491479.

16. Fujioka M, Munezawa T, Hayama T, Watanabe S, Kinoshita I, Ishida K, Satomi A, Takahashi K. "Intersigmoid hernia-report of two cases". Jpn J Gastroenterol Surg. 1976. Volume 9 Issue 2 Pages 199-203 DOI https://doi.org/10.5833/jjgs.9.199

17. Fukuzawa J, Matsuo R, Nakano Y. "A Case of Intersigmoid Hernia Treated with Laparoscopy". Nihon Gekakei Rengo Gakkaishi (Journal of Japanese College of Surgeons) 2014 Volume 39 Issue 2 223-227 DOI: https://doi.org/10.4030/jjcs.39.223

18. Furukawa k, Tsutsumi J, Takayama S *et al*. "A Case of Small Bowel Obstruction Due to Intramesosigmoid Hernia". Nihon Gekakei Rengo Gakkaishi (Journal of Japanese College of Surgeons). 2014 Volume 39 Issue 4 787-790 DOI https://doi.org/10.4030/jjcs.39.787

19. Ghahremani GG. "Abdominal and pelvic hernias". In: Gore RM, Levine MS, editors. Textbook of Gastrointestinal Radiology. Philadelphia, PA: Saunders; 2000. 1993–2009.

20. Guo L. "Diagnosis and treatment of congenital transmesosigmoid hernia". Yiyao Luntan Zazhi 2011; 32: 147-148

21. Harrison OJ, Sharma RD, Niayesh MH. "Early intervention in intersigmoid hernia may prevent bowel resection-A case report". Int J Surg Case Rep. 2011;2(8):282-4. doi: 10.1016/j.ijscr.2011.08.010. Epub 2011 Sep 14. PubMed PMID: 22096754; PubMed Central PMCID: PMC3215259.

22. Hattori S, Yamazaki S, Yoshida N *et al*. "A Case of Intramesosigmoid Hernia Invaginating into a Mesenteric Defect on the Right Peritoneal Surface of the Sigmoid Mesentery". Nihon Gekakei Rengo Gakkaishi (Journal of Japanese College of Surgeons). 2016 Volume 41 Issue 5 858-863 DOI https://doi.org/10.4030/jjcs.41.858

23. Hayama T, Shioya T, Hankyo M, Shimizu T, Shibuya H, Komine O, Watanabe Y, Nanbu K, Yamada T. "A Case of Laparoscopically Repositioned Intersigmoid Hernia in an 80s Man". Nihon Gekakei Rengo Gakkaishi (Journal of Japanese College of Surgeons). 2017 Volume 42 Issue 1 127-133 DOI https://doi.org/10.4030/jjcs.42.127

24. He M, Zhong QX, Yang ZX. "Congenital large transmesosigmoid hernia, case report". Zhengjiang Chuangshang Waike 1999; 7: 122

25. Hibino S, Sakakibara T, Niwa S, Oshima K, Ito, A, Sakakibara S. “A case of transmesosigmoid hernia”. Nihon Rinsho Geka Gakkai Zasshi (Journal of Japan Surgical Association). 2008 Volume 69 Issue 5 1274-1277 DOI: https://doi.org/10.3919/jjsa.69.1274
26. Hirashima K, Date K, Fujita K, Koide N, Kamuro A, Kato H, Fujita N. “Strangulation of the small intestine caused by an intra-mesosigmoid hernia: acase report”. Surg Case Rep. 2017 Dec 21;3(1):129. doi: 10.1186/s40792-017-0406-z.PubMed PMID: 29270809; PubMed Central PMCID: PMC5740056.
27. Hirata S, Yokoyama H, Otsu T *et al*. “A Case Report of Ileus due to Intramesosigmoid Hernia that Developed in the 29th Week of Pregnancy”. Nihon Fukubu Kyukyu Igakkai Zasshi (Journal of Abdominal Emergency Medicine). 2015 Volume 35 Issue 6 781-784 DOI: https://doi.org/10.11231/jaem.35.781
28. Hori Y, Nakamoto H, Itagaki R et al. “A Case of Laparoscope Assisted Repair for an Intramesosigmoid Hernia”. Nihon Fukubu Kyukyu Igakkai Zasshi (Journal of Abdominal Emergency Medicine). 2014 Volume 34 Issue 3 765-769 DOI: https://doi.org/10.11231/jaem.34.765
29. Horii N, Kamimukai N, Wada T *et al*. “A Case of Sigmoid Mesocolic Hernia”. Nihon Fukubu Kyukyu Igakkai Zasshi (Journal of Abdominal Emergency Medicine). 2015 Volume 35 Issue 1 131-134 DOI: https://doi.org/10.11231/jaem.35.131
30. Hoshikawa T, Oida Y, Nakamura T, Mukai M, Makuuchi H. “A case of internal hernia of the mesentery of the sigmoid colon”. Nihon Rinsho Geka Gakkai Zasshi (Journal of Japan Surgical Association). 2006 Volume 67 Issue 10 2474-2478 DOI: https://doi.org/10.3919/jjsa.67.2474
31. Ido M, Kuroda H, Ito A, Kato K, Kato H *et al*. “A case of intermesosigmoid hernia causing wide range of retroperitoneal abscess”. Nihon Rinsho Geka Gakkai Zasshi (Journal of Japan Surgical Association). 1998 Volume 59 Issue 12 3189-3193 DOI: https://doi.org/10.3919/jjsa.59.3189
32. Igarashi A., Okuda K., Nishiwaki M., Tsujitsuka K., Kiyono, T., Sumiyama M. “A Case of Internal Hernia Involving the Sigmoid Mesocolon Diagnosed Preoperatively”. Jpn J Gastroenterol Surg 31: 1816-1820, 1998
33. Ishita T, Oishi H, Ishii M *et al*. “A Case of Intersigmoid Hernia during Pregnancy”. Nihon Fukubu Kyukyu Igakkai Zasshi (Journal of Abdominal Emergency Medicine) 2015 Volume 35 Issue 3 289-292 DOI: https://doi.org/10.11231/jaem.35.289
34. Isobe H, Takiguchi M, Hayashi K, Miura T, Inaba Y, watabe S. “A case of intramesosigmoid hernia treated with laparoscopy”. Nihon Rinsho Geka Gakkai Zasshi (Journal of Japan Surgical Association). 2007 Volume 68 Issue 2 447-451 DOI: https://doi.org/10.3919/jjsa.68.44
35. Iwasaki K, Hujita T, Ohashi M. “A Case of Intramesosigmoid Hernia”. The Kitakanto Medical Journal 2016 Volume 66 Issue 2 163-166 DOI: https://doi.org/10.2974/kmj.66.163
36. Jimmy J, Wani SV, Shetty VV, Patankar RV. “Laparoscopic management of small bowel obstruction caused by a Sigmoid Mesocolic hernia. J Minim Access Surg. 2011 Oct;7(4):

236-8. doi: 10.4103/0972-9941.85647. PubMed PMID: 22022113; PubMed Central PMCID: PMC3193694.

37. Johnson BL, Lind JF, Ulich PJ. “Transmesosigmoid hernia during pregnancy”. South Med J. 1992 Jun;85(6):650-2. PubMed PMID: 1604396.
38. Kan H, Suzuki H, Takasaki H, Sasaki J, Furukawa K, Tajiri T. “A case of an intramesosigmoid hernia”. J Nippon Med Sch. 2009 Feb;76(1):13-8. PubMed PMID: 19305105.
39. Kaneko J, Imai N et al. “A Case Report of Transmesosigmoid Hernia”. The Japanese Journal of Gastroenterological Surgery. 1998 Volume 31 Issue 11 2280-2283 DOI: https://doi.org/10.5833/jjgs.31.2280
40. Katayanagi S, Ohue M, Yamaguchi T, Takahashi K, Mor T. “A Case Report of Intramesosigmoid Hernia”.The Japanese Journal of Gastroenterological Surgery. 2003 Volume 36 Issue 4 304-308 DOI: https://doi.org/10.5833/jjgs.36.304
41. Kayano H, Nomura E, Kuramoto T, Yatabe K, Yoshii H, Yokoyama D, Machida T, Uda S, Koike T, Izumi H, Hasegawa S, Mukai M, Makuuchi H. “Two Cases of Laparoscopic Diagnosis and Treatment of Intersigmoid Hernia”. Tokai J Exp Clin Med. 2017 Jul;42(2) 109-114. PMID: 28681372.
42. Kheok SW, Haja Mohideen SM, Lim JW, Santhosh Raj SR. “Intramesosigmoid hernia complicated by strangulated small-bowel obstruction”. BMJ Case Rep. 2016 Jul 15;2016. pii: bcr2016216369. doi: 10.1136/bcr-2016-216369. PubMed PMID: 27432828;PubMed Central PMCID: PMC4956957.
43. Kim, Ji Won, and Dae Hyun Yang. "Transmesosigmoid hernia with small bowel strangulation." International Journal of Case Reports and Images (IJCRI) 3.12 (2013): 25-28.
44. Kishi Y, Yumoto T, Tsukahara K, Nakao A, et al. (2017). “A Rare Presentation of Acute Abdomen due to Intersigmoid Hernia Successfully Treated by Laparoscopic Surgery. M J Surg 1(1): 005.
45. Kobayashi A, Koseki H, Masuko T, Owada Y. “Two Cases of Intestinal Obstruction Due to Internal Hernia”. The Japanese Journal of Gastroenterological Surgery 2000 Volume 33 Issue 5 634-638 DOI: https://doi.org/10.5833/jjgs.33.634
46. Kohga A, Iwasaki Y, Murata T, Okamoto J, Shimizu S, Takahashi S. “A case of intramesosigmoid hernia”. Nihon Rinsho Geka Gakkai Zasshi (Journal of Japan Surgical Association) 72(1):172-175 · January 2011 DOI https://doi.org/10.3919/jjsa.72.172
47. Koinuma J, Takeyama S, Sasamura Y, Hara T, Numata A, Nenohi M. A case of intramesosigmoid hernia diagnosed preoperatively and treated with laparoscopy. J Jpn Surg Assoc 2004; 65: 722-725.
48. Kojima S, Sakamoto T, Honda M, Kim DH. “Strangulated transmesosigmoid hernia as a late complication of a fall from a height: A case report. Int J Surg Case Rep. 2016;27:137-140. doi: 10.1016/j.ijscr.2016.08.031. Epub 2016 Aug 26. PubMed PMID: 27614337; PubMed Central PMCID:PMC5018078.

49. Koshino K, Kosugi C, Yasuda H, Koda K *et al*. "A Case of Internal Hernia of the Mesentery of the Sigmoid Colon". Nihon Fukubu Kyukyu Igakkai Zasshi (Journal of Abdominal Emergency Medicine). 2012 Volume 32 Issue 4 823-826 DOI: https://doi.org/10.11231/jaem.32.823
50. Kumagai K, Yoshioka M, Iida M, Kudoh K, Uchinami H, Watanabe T, Yamamoto Y. "Usefulness of Oblique Multiplanar Images on Multi-Detector Computed Tomography for Diagnosing Intersigmoid Hernia". Visc Med. 2016 Dec;32(6):437-440. Doi: 10.1159/000450633. Epub 2016 Nov 22. PubMed PMID: 28229081; PubMed Central PMCID: PMC5290440.
51. Kuroda T, Wakabayashi M *et al*. "A Case of Intramesosigmoid Hernia Treated by Laparoscopic Surgery". Journal of Nihon University Medical Association. 2015 Volume 74 Issue 5 263-266 DOI: https://doi.org/10.4264/numa.74.5_263
52. Li B, Assaf A, Gong YG, Feng LZ, Zheng XY, Wu CN. "Transmesosigmoid hernia: case report and review of literature. World J Gastroenterol. 2014 May 21;20(19):5924-9. doi: 10.3748/wjg.v20.i19.5924. Review. PubMed PMID: 24914355; PubMed Central PMCID: PMC4024804.
53. Li HC. Case report of transmesosigmoid hernia. Yishui Yizhuan Xuebao 1985; 7: 68
54. Luo ZK. "Misdiagnosed as intestinal necrosis of transmesosigmoid hernia, case report". Anhui Yike Daxue Xuebao 1996; 31: 202
55. Mandava, Rupesh, and Rajani Gorantla. "CT diagnosis of sigmoid." International Medical Journal 1.9 (2014): 548-550.
56. Martin LC, Merkle EM, Thompson WM. *"Review of internal hernias: radiographic and clinical findings"*. Am J Roentgenol. 2006; 186(3):703–17.
57. Maruyama H, Mio D, Takagi T, Kinoshita T, Nakayama H, Sekiya M. "A case of intersigmoid hernia treated with laparoscopic-assisted surgery". Nihon Rinsho Geka Gakkai Zasshi (Journal of Japan Surgical Association). 2005 Volume 66 Issue 8 2033-2037 DOI: https://doi.org/10.3919/jjsa.66.2033
58. Maruyama H, Suzuki N, Sasaki M, Sekiya M, Mio H. "Two cases of intersigmoid hernia". Jpn J Gastroenterol Surg. 2002; 35: 1721-1725.
59. Matsuda T, Morikawa E, Kubo R et al. "A case report of intersigmoid hernia". The Japanese Journal of Gastroenterological Surgery
60. Matsui T, Kitagami,H, Kondo Y, Nonoyama K, Watanabe K, Fujihata S, Yasuda A, Yamamoto M,Tanaka M. "An Intersigmoid Hernia Treated with Laparoscopic Surgery".The Japanese Journal of Gastroenterological Surgery. 2016 Volume 49 Issue 4 360-366 DOI: https://doi.org/10.5833/jjgs.2014.0271
61. Michiko Hinata, Mitsunori Saito and Tetsuya Aono. "Two Cases of Intramesosigmoid Hernia". Nihon Gekakei Rengo Gakkaishi (Journal of Japanese College of Surgeons). 2011 Volume 36 Issue 1 Pages 55-60 DOI https://doi.org/10.4030/jjcs.36.55

62. Mitsui K, Namiki K, Matsumoto H, Konno F, Takeyama D, Notsuda H. Laparoscopic treatment for intramesosigmoid hernia: report of a case. J Jpn Surg Assoc 2005; 66: 3006-3010.

63. Miura E, Sasaki S, Nakayama H, Watanabe T. “Three cases of intersigmoid hernia”. Nihon Rinsho Geka Gakkai Zasshi (Journal of Japan Surgical Association). 2013 Volume 74 Issue 3 838-843 DOI: https://doi.org/10.3919/jjsa.74.838

64. Mo J. “Case report of transmesosigmoid hernia causing small bowel necrosis”. Guangxi Yixue 1991; 18: 54

65. Moriyama H, Koyama T, Tanaka J, Terashima T, Kanou S. “A case of transmesosigmoid hernia”. Nihon Rinsho Geka Gakkai Zasshi (Journal of Japan Surgical Association). 2010 Volume 71 Issue 1 208-211 DOI: https://doi.org/10.3919/jjsa.71.208

66. Motohiro T, Sanada T, Omichi M, Hamada Y. “A Case of Transmesosigmoid Hernia in an Adult”. The Japanese Journal of Gastroenterological Surgery. 2004 Volume 37 Issue 11 1777-1780 DOI: https://doi.org/10.5833/jjgs.37.1777.

67. Murakami S. Okajima K, Isozaki H *et al*. “Intestinal obstruction due to intersigmoid hernia”. The journal of the Japanese Practical Surgeon Society 1996 Volume 57 Issue 8 1983-1987DOI https://doi.org/10.3919/ringe1963.57.1983

68. Murata Y, Miyata K, Yuasa N, Takeuchi E, Miyake H, Kobayashi Y. “A case of an intramesosigmoid hernia”. Nihon Rinsho Geka Gakkai Zasshi (Journal of Japan Surgical Association). 2009; Volume 70 Issue 3 886-890 doi: https://doi.org/10.3919/jjsa.70.886

69. Nachi S, Hino A, Iwata H, Sekino T. “A case of intramesosigmoid hernia with summary of the Japanese literature”. Nihon Rinsho Geka Gakkai Zasshi (Journal of Japan Surgical Association). 2005 volume 66, No. 11 2807-2811 DOI https://doi.org/10.3919/jjsa.66.2807

70. Nagano H, Goi T, Taguchi S, Tsubaki T, Uematsu H. “Diagnosis of incarcerated intramesosigmoid hernia aided by multiplanar reconstruction images of multidetector computed tomography: a case report”. Surg Case Rep. 2018;4(1):128. Published 2018 Oct 20. doi:10.1186/s40792-018-0535-z

71. Nihon-Yanagi Y, Ooshiro M, Osamura A, Takagi R, Moriyama A, Urita T, Yoshida Y, Tanaka H, Sugishita Y, Oshiro T, Kinoshita T, Nagashima M, Okazumi S. “Intersigmoid hernia: Report of a case”. Surg Today. 2010;40(2):171-5. doi: 10.1007/s00595-009-4010-0. Epub 2010 Jan 28. Review. PubMed PMID: 20107960.

72. Nishida Y, Takahashi Y, Sasahara K. “Laparoscopically Treated Strangulated Ileus Due to an Intersigmoid Hernia”. Nihon Fukubu Kyukyu Igakkai Zasshi (Journal of Abdominal Emergency Medicine). 2013 Volume 33 Issue 1 91-94 DOI: https://doi.org/10.11231/jaem.33.91

73. Nitta H, Ishikawa F, Omoto H, Yamashita S, Suwa T. “Laparoscopic treatment for intramesosigmoid hernia-a case report”. Nihon Rinsho Geka Gakkai Zasshi (Journal of

Japan Surgical Association). 2006 Volume 67 Issue 10 2479-2484 DOI: https://doi.org/10.3919/jjsa.67.2479

74. Okada A, Yokota K, Itakura Y *et al*. “A case of intersigmoid hernia with characteristic computed tomography findings”. Nihon Rinsho Geka Gakkai Zasshi (Journal of Japan Surgical Association). 2010 Volume 71 Issue 6 1624-1627 DOI: https://doi.org/10.3919/jjsa.71.1624
75. Okutani D, Shien T, Soh J, Udaka T. “A case of strangulated ileus due to intramesosigmoid hernia”. Nihon Rinsho Geka Gakkai Zasshi (Journal of Japan Surgical Association). 2001 Volume 62 Issue 3 817-820 DOI: https://doi.org/10.3919/jjsa.62.3_817
76. Oshima R, Minato E, Sakurau J *et al*. “A case of transmesosigmoid hernia”. Nihon Rinsho Geka Gakkai Zasshi (Journal of Japan Surgical Association). 2010 Volume 71 Issue 3 839-843 DOI: https://doi.org/10.3919/jjsa.71.839
77. Ota H, Yamazaki, Endoh W, Hojo S, Ueda N, Maeura Y. “A case of intramesosigmoid hernia invaginating into a mesenteric defect of the left peritoneal surface of the sigmoid mesentery”. Nihon Rinsho Geka Gakkai Zasshi (Journal of Japan Surgical Association). 2005 Volume 66 Issue 3 770-773 DOI: https://doi.org/10.3919/jjsa.66.770
78. Papanikolaou G, Alexiou GA, Mitsis M, Markouizos G, Kappas AM. “Congenital transmesosigmoid hernia: a rare case of pediatric small-bowel obstruction”. Pediatr Emerg Care. 2008 Jul;24(7):471-3. doi: 10.1097/PEC.0b013e31817de2a4. PubMed PMID: 18633309.
79. Perez Rouiz L, Gabarrell Oto A, Casals Garrigo R, Sola Marti R, Ziza F. “Intestinal obstruction caused by internal transmesosigmoid hernia: a complication of laparoscopic surgery?”. Minerva Chir. 1997 Sep;52(9):1109-12. Italian. PubMed PMID: 9432585.
80. Reddy, N. V. N., and Sunil Koushik. "A rare cause of internal herniation-Intermesocolic fossa of Brosike." Journal of Medical & Allied Sciences 5.1 (2015): 18.
81. Sasaki T, Sakai K, Fukumori D, Sato M, Ohmori H, Yamamoto F. “Transmesosigmoid hernia: report of a case”. Surg Today. 2002;32(12):1096-8. PubMed PMID: 12541031.
82. Sasaki Y, Akashi S, Sugimori S, Yamada Y. “A Case of Transmesosigmoid Hernia Preoperatively Diagnosed”. Nihon Gekakei Rengo Gakkaishi (Journal of Japanese College of Surgeons). 2017 Volume 42 Issue 1 122-126 DOI: https://doi.org/10.4030/jjcs.42.122
83. Satomura H, Masuda N, Takita J, Mogami Y, Kato H “Two cases of intramesosigmoid hernia performed laparoscopic treatment”. Nihon Rinsho Geka Gakkai Zasshi (Journal of Japan Surgical Association) 72(7):1870-1876 · January 2011 DOI: 10.3919/jjsa.72.1870
84. Sawazaki S, Hayashi S,Tsuchida K *et al*. “A Case of Intramesosigmoid Hernia Diagnosed with Laparoscopy”. Nihon Gekakei Rengo Gakkaishi (Journal of Japanese College of Surgeons). 2012 Volume 37 Issue 6 1221-1225 DOI: https://doi.org/10.4030/jjcs.37.1221
85. Saxena, D., Shah, Y., Sahu, D., Sonarkar, R., Tandon M. “Strangulated Transmesosigmoid Hernia: A Rare Cause of Acute Abdomen”. Euroasian J Hepato-Gastroenterol 2013;3(2): 139-141 doi: 10.5005/jp-journals-10018-1085

86. Shibahara K, Nakada K, Watanabe T, Sasaki M. “A case of intrasigmoid hernia”. Nihon Rinsho Geka Gakkai Zasshi (Journal of Japan Surgical Association). 1999 Volume 60 Issue 7 1926-1929 DOI: https://doi.org/10.3919/jjsa.60.1926
87. Shibuya N, Ishizuka M, Iwasaki Y, Takagi K, Nagata H, Aoki T, Kubota K. “Usefulness of a laparoscopic approach for treatment of small-bowel obstructiondue to intersigmoid hernia: a case report”. Surg Case Rep. 2017 Dec;3(1):22. doi: 10.1186/s40792-017-0298-y. Epub 2017 Feb 4. PubMed PMID: 28161873; PubMed CentralPMCID: PMC5292108.
88. Shinoto K, Oshima I, Ariga T, Ozaki M. “A Case of Transmesosigmoid Hernia Diagnosed as Strangulated Ileus”. Nihon Fukubu Kyukyu Igakkai Zasshi (Journal of Abdominal Emergency Medicine). 2012 Volume 32 Issue 3 715-718 DOI: https://doi.org/10.11231/jaem.32.715
89. Shinto O, Kim E, Takatsuka S, Yui S, Ikehara T. “A case of intramesosigmoid hernia”. Nihon Rinsho Geka Gakkai Zasshi (Journal of Japan Surgical Association). 2005 Volume 66 Issue 4 871-875 DOI: https://doi.org/10.3919/jjsa.66.871
90. Siaperas P., Vallas Ch., Karanikas I. and Oikonomou N. “Incarcerated Transmesosigmoid Hernia Presenting in a 28-Year-Old Female”Hellenic Journal of Surgery, 2014, Volume 86, Number 3, Page 186 DOI: 10.1007/s13126-014-0125-x
91. Sogo Y, Fujiwara H, Yamasaki M, Okamoto T, Sakakua S, Tomiyoshi H, Ohyanagi H. “A case of intramesosigmoid hernia”. The Japanese Journal of Gastroenterological Surgery 2001 Volume 34 Issue 9 1461-1465 DOI: https://doi.org/10.5833/jjgs.34.1461
92. Steele G Jr, Sawyer RB, Sawyer KC. “Retroperitoneal and transmesosigmoid herniation of ileum. Report of a case”. Rocky Mt Med J. 1973 Jun;70(6):30-2. PubMed PMID: 4710725.
93. Sugimoto I, Kato R, Kazui H, Kitagawa S, Imamura Y, Hasegawa T, et al. “A case report of intersigmoid hernia”. J. Aichi Med. Univ. Assoc. 1985; 13: 242-245
94. Tada M, Kanamaru H, Horie T, Odaka A Murata M. “A Case Report of Intrasigmoid Hernia”. The Japanese Journal of Gastroenterological Surgery. 1994 Volume 27 Issue 12 2605-2608 DOI: https://doi.org/10.5833/jjgs.27.2605
95. Takayanagi D, Mazaki T, Madou K, Ogame H, Ishii Y, Masuda H. “A case of intramesosigmoid hernia”. Nihon Rinsho Geka Gakkai Zasshi (Journal of Japan Surgical Association). 2010 Volume 71 Issue 6 1619-1623 DOI https://doi.org/10.3919/jjsa.71.1619
96. Takeshita T, Ninoi T, Shigeoka H, Hirayama Y, Miki Y. “Small bowel obstruction due to an intramesosigmoid hernia diagnosed by multidetector row computed tomography: a case report”. Osaka City Med J. 2010 Dec;56(2):37-45. PubMed PMID: 21466128.
97. Takikawa T, Negishi K, Kato T, Yamasaki H, Hattori M. “A case of internal hernia of the mesentery of the sigmoid colon presented with ileus”. 2001 Volume 62 Issue 11 2819-2822 DOI: https://doi.org/10.3919/jjsa.62.281

98. Tanaka K, Yokoyama H, Hirata S *et al*. "An Emergency Operation in a Case of Ileus due to an Intersigmoid Hernia". Nihon Fukubu Kyukyu Igakkai Zasshi (Journal of Abdominal Emergency Medicine). 2014 Volume 34 Issue 7 1395-1399 DOI: https://doi.org/10.11231/jaem.34.1395
99. Taniguchi K, Iida R, Ota K, Asakuma M, Uchiyama K, Takasu A. "Single-port surgery (SPS) strategy for small bowel obstruction (SBO) caused by postoperative internal hernia: A series case report". Medicine (Baltimore). 2018 Mar;97(13): e0269. doi: 10.1097/MD.0000000000010269. PubMed PMID: 29595689; PubMed Central PMCID: PMC5895389.
100. Teruya T, Takaesu Y, Hokama A, Nohara M, Muto Y. "Intramesosigmoid Hernia-a case report". Nihon Rinsho Geka Gakkai Zasshi (Journal of Japan Surgical Association). 1998 Volume 59 Issue 5 1401-1404 DOI https://doi.org/10.3919/jjsa.59.1401
101. Toyoda S, Ichikawa T, Imagawa A *et al*. "Two Cases of Intersigmoid Hernia Treated with Laparoscopic Surgery". Nihon Gekakei Rengo Gakkaishi (Journal of Japanese College of Surgeons). 2013 Volume 38 Issue 1 178-183 DOI: https://doi.org/10.4030/jjcs.38.178
102. Tsukahara T, Yamaguchi A, Isogai M *et al*. "Two cases of intersigmoid hernia diagnosed preoperatively". Nihon Rinsho Geka Gakkai Zasshi (Journal of Japan Surgical Association). 2008 Volume 69 Issue 3 676-681 DOI: https://doi.org/10.3919/jjsa.69.676
103. Van der Mieren G, de Gheldere C, Vanclooster P. "Transmesosigmoid hernia: report of a case and review of the literature". Acta Chir Belg. 2005 Nov-Dec;105(6):653-5. Review. PubMed PMID: 16438079.
104. Waku T, Watanabe K, Tomioka N, Orita Y. "Two Cases of Incarcerated Internal Hernia". The Japanese Journal of Gastroenterological Surgery. 2005 Volume 38 Issue 5 545-548 DOI: https://doi.org/10.5833/jjgs.38.545
105. Watanabe M, Mitsumori N, Shida A *et al*. "A Case Report of Intramesosigmoid Hernia Treated with Laparoscopy". The Japanese Journal of Gastroenterological Surgery. 2003 Volume 36 Issue 4 309-313 DOI: https://doi.org/10.5833/jjgs.36.309
106. Watanabe M, Morioka J. "A Case of Intramesosigmoid Hernia Diagnosed by Computed Tomography and Treated with Laparoscope". Nihon Gekakei Rengo Gakkaishi (Journal of Japanese College of Surgeons). 2015 Volume 40 Issue 6 1189-1194 DOI: https://doi.org/10.4030/jjcs.40.1189
107. Watanabe T, Wada H, Sato M, Miyaki Y, Shiiya N. "Single-incision laparoscopic surgery for intersigmoid hernia". Case Rep Surg. 2014;2014:589649. doi: 10.1155/2014/589649. Epub 2014 Nov 19. PubMed PMID: 25506032; PubMed Central PMCID: PMC4258357.
108. Yang C, Kim D. "Small bowel obstruction caused by sigmoid mesocolic hernia". J Surg Case Rep. 2014 May 2;2014(5). pii: rju036. doi: 10.1093/jscr/rju036. Print 2014 May. PubMed PMID: 24876507; PubMed Central PMCID: PMC4207257.
109. Yang MS, Yeh DM, Lin SS, Chang CC, Wu MM, Chao C, Tyan YS. "Computed tomographic appearance of internal herniation through the sigmoid mesocolon". J Chin Med Assoc. 2005 Apr;68(4):195-7 PubMed PMID: 15850072.
110. Yang WL, Zhang DW, Zhang HG. "Diagnosis and treatment of adult congenital transmesentery hernia". Zhongguo Weichang Waike Zazhi 2010; 13: 621-622

111. Yao YF, Zhang MF. “Ultrasonic manifestations of congenital transmesosigmoid hernia: report of one case”. Zhongguo Linchang Yixue Yingxiang Zazhi 2012; 23: 149-150
112. Yazawa T, Shimizu T, Mekata E, Sonoda H, Tatsuta T, Tani T. “A case of intersigmoid hernia diagnosed preoperatively”. Nihon Rinsho Geka Gakkai Zasshi (Journal of Japan Surgical Association) 2011 Volume 72 Issue 10 2676-2680 DOI: https://doi.org/10.3919/jjsa.72.2676
113. Yu CY, Lin CC, Yu JC, Liu CH, Shyu RY, Chen CY. “Strangulated transmesosigmoid hernia: CT diagnosis”. Abdom Imaging. 2004 Mar-Apr;29(2):158-60. PubMed PMID: 15290938.
114. Zhang JS, Luo RH, Gao XY. “Congenital transmesosigmoid hernia causing small bowel strangulation, case report”. Binzhou Yixueyuan Xuebao 1995; 5: 46
115. Zhou FZ, Zhou ZQ, Chen HL, Chen DH, Chen WM, Wu YP. “Five cases of mesentery defect causing hernia in schizophrenia patients”. Zhengjiang Chuangshang Waike 2007; 12: 272-273

SDC 2. Table 2: Characteristics of included studies.

References	Imaging before operation	Abdominal X-ray Contrast study of SI	Abdominal CT	MDCT	Abdominal US
Benson 1964	AXR	"distention of two loops of SB with air-fluid levels"	NP	NP	NP
Steele 1973	AXR	"dilated SBL with multiple air-fluid levels"	NP	NP	NP
Fujioka 1976	AXR	NR	NP	NP	NP
Bircher 1981	AXR	"Multiple air fluid levels, dilated SB"	NP	NP	NP
Li 1982	AXR	"Multiple air fluid levels, dilated SBL"	NP	NP	NP
Li 1985	AXR	"Multiple air fluid levels, dilated SBL"	NP	NP	NP
Sugimoto 1985	AXR	"air and fluid levels in SB"	NP	NP	NP
Matsuda 1989	AXR	"distended SB with air-fluid levels"	NP	NP	NP
Mo 1991	AXR	"Multiple, dilated SBL"	NP	NP	NP

Johnson 1992	AXR	"dilated SBL with multiple air-fluid levels"	NP	NP	NP
Tada 1994	AXR	"Distended SB with air-fluid levels"	NP	NP	NP
Zhang 1995	AXR	"Multiple air fluid levels, dilated SBL"	NP	NP	NP
Luo 1996	AXR	"Multiple niveau with dilated SBL"	NP	NP	NP
Murakami 1996	AXR	"Dilated SBL"	NP	NP	NP
Perez 1997	AXR, US	"dilated SB with multiple air fluid levels"	NP	NP	"dilated SBL with free intraperitoneal liquid"
Ido 1998	AXR, ACT	"dilated SI with air-fluid levels"	"SI obstraction and retroperitoneal abscess"	NP	NP
Igarashi 1998	CCSI, ACT	"air and fluid levels in SB"	"stenotic portion of the ileum in the sigmoid mesocolon"	NP	NP
Kaneko 1998	AXR, ACT	"dilatation of SI gas image"	"Dilated SBL"	NP	NP
Teruya 1998	AXR, ACT	"Dilated SBL"	"Dilated SBL"	NP	NP
He 1999	AXR	"Multiple air-fluid levels"	NP	NP	NP

Shibahara 1999	AXR, ACT	"niveau in the SI"	"dilated SB"	NP	NP
Kobayashi 2000	AXR, CSSI	"Diffuse SB gas with air-fluid, smooth narrowing of SB"	NP	NP	NP
Endo 2001	CSSI, ACT	"stenosis"	"SB included in the SM"	NP	NP
Okutani 2001	AXR, ACT	"multiple niveau"	"Ascites and dilatation of SI"	NP	NP
Sogo 2001	AXR, CSSI	" air fluid levels, stenosis"	NP	NP	NP
Takikawa 2001	AXR, ACT	"air-fluid levels"	"intussusception of the SI"	NP	NP
Maruyama 2002	AXR	" SI gas and fluid"	NP	NP	NP
Sasaki 2002	AXR, ACT	"distended SBL and niveau formation"	"very dilated SI loops with obstruction in the terminal ileum"	NP	NP
Katayanagi 2003	AXR, CSSI, ACT	"SB gas with air-fluid" "2 smooth narrowing"	"dilatation and stenosis of SB"	NP	NP
Watanabe 2003	AXR	"Dilated SI, air-fluid levels"	NP	NP	NP
Akiyama 2004	AXR, CSSI, ACT	"SB gas, stenosis"	"dilated SI at the pelvic space"	NP	NP

Koinuma 2004	CSSI, ACT	"dilatation and stenosis"	"small bowel wall was partially thickened"	NP	NP
Motohiro 2004	AXR, ACT, AUS	"dilatation and gas in the SI"	"small bowel situated in the SM"	NP	"Ascites, keyboard sign"
Yu 2004	ACT	NR	"A cluster of dilated, fluid-filled, U- and C-shaped loops of SB"	NP	NP
Maruyama 2005	AXR, CSSI, ACT	"Air- fluid levels, stenosis"	"Ileum stenosis in the pelvis"	NP	NP
Mitsui 2005	ACT	NP	" incarcerated SBL"	NP	NP
Nachi 2005	AXR, ACT	"Ileus"	"tapered obstruction of SI in the left side of lower abdomen"	NP	NP
Ota 2005	AXR, ACT	"Multiple niveaus of the SB"	"Dilated SB"	NP	NP
Shinto 2005	AXR, ACT	"SI gas with air-fluid levels"	"Dilated SBL"	NP	NP
Van der Mieren 2005	AXR, ACT	NR	NR	NP	NP
Waku 2005	CSSI, AXR	"Obstruction of SI at the SM"	"Obstruction of SI at the lower abdomen"	NP	NP
Yang 2005	ACT	NP	"A short segment of SBL with thickened and edematous wall at the left-lower abdomen"	NP	NP

Hoshikawa 2006	AXR, ACT	"Gas in the SI"	"remarkable dilatation of the entire SI"	NP	NP
Nitta 2006	AXR, ACT	"Dilated SB"	"dilatation of SB"	NP	NP
Isobe 2007	AXR, CSSI, ACT	"Dilated and stenotic SB"	"Dilated SB"	NP	NP
Zhou 2007	ACT	NP	"dilated SBL and collapsed distal SB"	NP	NP
Collins 2008	AXR, ACT	"two prominent loops of SB in the right lower quadrant"	"dilated loops of SB with a zone of transition approximately 10 cm from the ileo-caecal valve"	NP	NP
Hibino 2008	AXR, ACT	"Dilated SB"	"Ascites and dilated SBL"	NP	NP
Papanikolaou 2008	US	NP	NP	NP	"dilated SB with free intraperitoneal liquid"
Tsukahara 2008	CSSI, ACT<br>ACT	"Stenosis at the ileum"<br>NP	"Incarcerated SBL in sigmoid mesocolon"<br>" expanded discontinuous SB around the SM"	NP	NP
Bao SQ 2009	CT	NP	"dilated SB and collapsed distal SB"	NP	NP
Kan 2009	AXR, ACT	"SB gas with niveau formation"	"2 strictures in SB separated by approximately 10 cm"	NP	NP
Murata 2009	ACT	NP	"dilated SB and collapsed distal SB"	NP	NP

Moriyama 2010	ACT	NP	"SB dilated and ascites"	NP	NP
Nihon-yanagi 2010	AXR, ACT	"Prominent SB gas"	"wall thickening of the edematous, fluid-filled ileum in the left iliac fossa and pelvic cavity"	NP	NP
Okada 2010	AXR, CSSI, ACT	"Dilated SB, niveau formation"	"Dilated SI in the left side of SM"	NP	NP
Oshima 2010	AXR, ACT	"Dilated SI, air-fluid levels"	SBL with poorly enhanced wall"	NP	NP
Takayanagi 2010	AXR, ACT	"dilated SB, multiple niveau"	"dilated SB and a stenotic portion"	NP	NP
Takeshita 2010	AXR, ACT	"Dilatation and an air-fluid level of SB"	NP	"dilated SBL, a sac-like mass "	NP
Yang WL 2010	CT	NR	"dilated SBL and collapsed distal SB"	NR	NR
Agarwal 2011	AXR	"Multiple air-fluid levels"	NP	NP	NP
Guo 2011	CT	NP	"ascites, dilated SBL and intestinal wall tickening"	NP	NP
Harrison 2011	AXR, ACT	"dilated stomach and SB loops"	"dilated loops of proximal SB with normal distal SB"	NP	NP
Jimmy 2011	AXR, ACT	"Single dilated loop of SB in the left lower abdomen"	"Dilated SBL with a cut-off at the level of mid-ileum"	NP	NP

Kohga 2011	ACT	NP	“bird-beak sign in the lower left quadrant with proximal intestine dilatation”	NP	NP
Michiko 2011	AXR, ACT	“multiple air fluid levels”<br>“multiple air fluid levels”	“SI obstruction of the left lower quadrant”<br>“SI obstruction in the pelvic cavity”	NP<br>NP	NP<br>NP
Satomura 2011	AXR, ACT<br><br>AXR, ACT	“ dilated SI and a few colon gas”<br>“ SB gas and a few colon gas”	“SI has been incarcerated like “eight figure” behind the mesentery of sigmoid colon”<br>“SI has been incarcerated like “eight figure” behind the mesentery of sigmoid colon”	NP<br><br>NP	NP<br><br>NP
Yazawa 2011	AXR, ACT	“SI gas”	“closed loop of the SI in sigmoid colon”	NP	NP
Kim 2012	AXR, ACT	“mild ileus without prominent dilation of SB”	“ascites, dilated SBL and intestinal wall tickening”	NP	NP
Koshino 2012	AXR, ACT	“SI dilated”	“Dilatation of SI”	NP	NP
Sawazaki 2012	AXR, ACT	“dilatation and stenosis”	“dilatation and stenosis of SB”	NP	NP
Shinoto 2012	AXR, CSSI, ACT	NR	“incarcerated SBL in the lateral position of SM”	NP	NP
Yao 2012	US	NP	NP	NP	“Dilated SBL”
Miura 2013	AXR, ACT<br>AXR, ACT<br>AXR, ACT	“Multiple niveau in SI”<br>“Gas in the SI”<br>Gas in the SI”	“dilated and thickened SI, beak sign”<br>“dilated and thickened SI”<br>“distended SI in the SM, closed loop in LLQ”	NP<br>NP<br>NP	NP<br>NP<br>NP

Nishida 2013	ACT	NP	“Loop-shaped small bowel in LLQ”	NP	NP
Saxena 2013	AXR, US	“dilated SB loops with multiple air fluid levels”	NP	NP	“Dilated SBL”
Toyoda 2013	AXR, ACT, AUS AXR, ACT	“dilated SI” “Air-fluid levels in the SB”	“cystic mass, dilation and narrowing of the SB” “closed loop an area of narrowing of the SB”	NP NP	“ Cystic mass in LLQ” NP
Bandawar 2014	AXR	“dilated SB loops with multiple air fluid levels”	NP	NP	NP
Fukuzawa 2014	AXR, CSSI, ACT	“dilated SI with niveau”	Dilated SI and obstruction of SI in the SM”	NP	NP
Furukawa 2014	CCSI, ACT	“dilated SB and stenosis”	“ascites around the liver and incarcerated SB”	NP	NP
Hori 2014	AXR, ACT	“Dilated SB, niveau”	“localized SI dilation ait the dorsal of SM”	NP	NP
Li 2014	ACT	NP	“dilated SBL with multiple air fluid levels and collapsed distal SB”	NP	NP
Mandava 2014	AXR, ACT	“Multiple niveau, dilated SB”	“Dilated SBL with bird beak appearance”	NP	NP
Siaperas 2014	AXR, ACT	“Dilatation and an air-fluid level of SB”	“distended stomach and SBL with the presence of fluid in the lesser pelvis”	NP	NP
Tanaka 2014	AXR, ACT	“niveau in the SI”	“ascites, dilation of the SI with a closed loop at the dorsal surface of SM”	NP	NP

Watanabe 2014	AXR, ACT	"Dilatation and an air-fluid level of SB"	"Extensively dilated SB and a closed loop of SB in the mesosigmoid"	NP	NP
Yang 2014	AXR, ACT	"slight distension of SB with air-fluid level"	"dilated SBL"	NP	NP
Campbell 2015	AXR, ACT	"Multiple dilated loops of SB"	"a dilated proximal SB with a transition point in the left iliac fossa"	NP	NP
Farah 2015	AXR, ACT	"2 prominent loops of SB in the left lower quadrant"	"SI dilatation with free fluid level"	NP	NP
Hirata 2015	AXR, ACT	NR	NR	NP	NP
Horii 2015	AXR, ACT	"small mass in LLQ"	"dilated loops of SI, ascetic fluid in pelvis"	NP	NP
Ishita 2015	AXR, ACT	"dilatation of SI"	"enlargement of the SI and ascites"	NP	NP
Kuroda 2015	ACT	NP	"dilatation of SI and stenosis of pelvic tract"	NP	NP
Reddy 2015	AXR	"Multiple air fluid levels on the right lower abdomen"	NP	NP	NP
Shukla 2015	AXR, ACT	"Multiple air fluid levels and dilated SB loops"	"dilated SBL with minimal interloop fluid"	NP	NP
Watanabe 2015	ACT	NP	"incarcerated intestine like a 8 figure"	NP	NP

Benyamini 2016	ACT	NP	"Mildly dilated thin-walled small bowel in the mid-abdomen and pelvis"	NP	NP
Hattori 2016	AXR, ACT	"a distended SB"	"loop of the SI with proximal dilatation"	NP	NP
Iwasaki 2016	ACT	"dilation and local narrowing in the left lower quadrant"	NP	NP	NP
Kheok 2016	AXR, ACT	"Dilation of SBL and multiple air fluid levels"	"C-shaped appearance of the dilated SB with mesenteric engorgement and fat stranding"	NP	NP
Kojima 2016	AXR, ACT	"Dilatation and an air-fluid level of SB"	extensively dilated SB and a closed loop of small bowel in the pelvis, with no signs of adhesion	NP	NP
Kumagai 2016	AXR, ACT, MDCT	"Diffuse dilatation of the SB"	"dilated SBL"	"narrowing and wall thickening of SI"	NP
Matsui 2016	AXR, ACT	"dilated SB"	"stenosis of SI in the left lower abdomen"	NP	NP
Agatsuma 2017	ACT	NP	" dilation SI, bowel wall thickening and the closed loop sign"	NP	NP
Ahmad 2017	AXR	"4-5 air-fluid levels without much bowel dilatation"	NP	NP	NP

Chou 2017	ACT	NP	"long small bowel segment traversing dorsal to the sigmoid colon to left lower abdomen"	NP	NP
Hayama 2017	AXR, ACT	"dilatation of SI with niveau"	"dilation of SI"	NP	NP
Hirashima 2017	AXR, ACT	"A large amount of gas in the SI"	"Stenosis of SI in the LLQ, dilation on the dorsal side of the sigmoid colon"	NP	NP
Kayano 2017	AXR, ACT<br><br>AXR, ACT	"A large amount of gas in small intestine"<br><br>"Dilatation of the SI with multiple niveau"	"Stenosis of SI in the left lower abdomen and dilation on the dorsal side of the sigmoid colon"<br><br>"SI extending in the shape of the figure 8 in the left lower abdomen"	NP<br><br>NP	NP<br><br>NP
Kishi 2017	AXR, ACT	NR	"wall thickening of the edematous, fluid-filled ileum in the left iliac fossa and pelvic cavity"	NP	NP
Sasaki 2017	AXR, ACT	Dilatation of SB"	"Dilatation of SI herniated in the SM"	NP	NP
Shibuya 2017	AXR, ACT	"Dilatation of SB"	"obstruction of the SB in the left lower abdomen with SB dilatation on the oral side"	NP	NP
Nagano 2018	MDCT	NP	NP	" incarcerated SBL in the left pelvic space"	NP
Taniguchi 2018	ACT	NP	"A closed loop obstruction around sigmoid colon"	NP	NP

SDC 3. Table 3: Radiological evaluation of included studies.

References	Duration of the procedure	Diameter of defect (cm)	Length of incarcerated small bowel (cm)	Adhesions of herniated ileum at the hernial orifice	Type of adhesion
Benson 1964	NR	2	15	NR	NR
Steele 1973	NR	2	NR	NR	NR
Fujioka 1976	NR<br>NR	2,5<br>1,5	3<br>5	NR<br>NR	NR<br>NR
Bircher 1981	NR<br>NR	NR<br>NR	NR<br>NR	NR<br>NR	NR<br>NR
Li 1982	NR	4	NR	NR	NR
Li 1985	NR	2	NR	NR	NR
Sugimoto 1985	NR	3	15	NR	NR
Matsuda 1989	NR	3	10	NR	NR
Mo 1991	NR	3	NR	NR	NR
Johnson 1992	NR	5	NR	NR	NR
Tada 1994	NR	2	10	NR	NR
Zhang 1995	NR	3	NR	NR	NR
Luo 1996	NR	3	NR	NR	NR
Murakami 1996	NR	1,5	5	NR	NR

Perez 1997	NR	NR	NR	NR	NR
Ido 1998	NR	1	NR	NR	NR
Igarashi 1998	NR	2,1	7,5	NR	NR
Kaneko 1998	NR	2,5	150	NR	NR
Teruya 1998	NR	1,5	10	NR	NR
He 1999	NR	3	NR	NR	NR
Shibahara 1999	NR	1,5	NR	NR	NR
Kobayashi 2000	NR	2<br>2	20<br>10	Yes<br>No	Mild<br>NR
Endo 2001	NR	2	130	NR	NR
Okutani 2001	NR	10	70	NR	NR
Sogo 2001	NR	3	NR	NR	NR
Takikawa 2001	NR	2	70	NR	NR
Maruyama 2002	NR<br>NR	3<br>2,5	5<br>5	NR<br>NR	NR<br>NR
Sasaki 2002	NR	3,5	30	NR	NR
Katayanagi 2003	NR	3	15	NR	NR
Watanabe 2003	NR	NR	10	NR	NR
Akiyama 2004	NR	2	4	NR	NR
Koinuma 2004	NR	2	NR	NR	NR

Motohiro 2004	NR	5	50	NR	NR
Yu 2004	NR	5	NR	NR	NR
Maruyama 2005	NR	2,5	NR	NR	NR
Mitsui 2005	NR	3	15	NR	NR
Nachi 2005	NR	2,5	10	NR	NR
Ota 2005	NR	3	NR	NR	NR
Shinto 2005	NR	2	NR	NR	NR
Van der Mieren 2005	NR	NR	NR	NR	NR
Waku 2005	NR	2,5	NR	NR	NR
Yang 2005	NR	NR	20	NR	NR
Hoshikawa 2006	NR	2	NR	NR	NR
Nitta 2006	NR	2,5	NR	NR	NR
Isobe 2007	NR	3	4	NR	NR
Zhou 2007	NR	3	NR	NR	NR
Collins 2008	NR	4	NR	NR	NR
Hibino 2008	NR	3	40	NR	NR
Papanikolaou 2008	NR	5	NR	NR	NR
Tsukahara 2008	NR<br>NR	3<br>2	10<br>20	NR<br>NR	NR<br>NR

Bao SQ 2009	NR	3	NR	NR	NR
Kan 2009	NR	4	NR	NR	NR
Murata 2009	NR	2	4	NR	NR
Moriyama 2010	NR	2	15	NR	NR
Nihon-Yanagi 2010	NR	3	NR	Yes	NR
Okada 2010	NR	1,5	10	NR	NR
Oshima 2010	NR	1	10	NR	NR
Takayanagi 2010	NR	2	NR	NR	NR
Takeshita 2010	NR	2,5	20	NR	NR
Yang WL 2010	NR<br>NR	5<br>4	NR<br>NR	NR<br>NR	NR<br>NR
Agarwal 2011	NR	1,8	15	NR	NR
Guo 2011	NR	4	NR	NR	NR
Harrison 2011	NR	NR	NR	NR	NR
Jimmy 2011	NR	2	NR	NR	NR
Kohga 2011	NR	3	NR	NR	NR
Michiko 2011	NR<br>NR	1,5<br>2	7<br>NR	NR<br>NR	NR<br>NR
Satomura 2011	65 minutes<br>59 minutes	4<br>3	8<br>10	NR<br>NR	NR<br>NR

Yazawa 2011	NR	1,5	10	No	NR
Kim 2012	NR	3	150	Yes	Mild
Koshino 2012	NR	2	10	NR	NR
Sawazaki 2012	NR	2	10	Yes	Mild
Shinoto 2012	NR	2	150	NR	NR
Yao 2012	NR	4	NR	NR	NR
Miura 2013	NR<br>NR<br>NR	2<br>3<br>1,5	NR<br>NR<br>15	NR<br>NR<br>NR	NR<br>NR<br>NR
Nishida 2013	NR	2	10	NR	NR
Saxena 2013	NR	4	50	NR	NR
Toyoda 2013	NR<br>NR	2<br>1	20<br>75	NR<br>NR	NR<br>NR
Bandawar 2014	NR	2	25	NR	NR
Fukuzawa 2014	44 minutes	NR	NR	NR	NR
Furukawa 2014	NR	2	10	NR	NR
Hori 2014	NR	3	NR	NR	NR
Li 2014	40 minutes	2,5	15	NR	NR
Mandava 2014	NR	NR	NR	NR	NR
Siaperas 2014	NR	3	NR	NR	NR

Tanaka 2014	NR	2	5	Yes	NR
Watanabe 2014	NR	3	10	Yes	Mild
Yang 2014	NR	2	15	NR	NR
Campbel 2015	60 minutes	3	6	NR	NR
Farah 2015	NR	4,5	NR	NR	NR
Hirata 2015	NR	2	NR	NR	NR
Horii 2015	NR	2	30	NR	NR
Ishita 2015	NR	2	NR	NR	NR
Kuroda 2015	NR	3	10	NR	NR
Reddy 2015	NR	NR	NR	NR	NR
Shukla 2015	NR	2	NR	NR	NR
Watanabe 2015	NR	3	15	NR	NR
Benyamini 2016	NR	2	NR	NR	NR
Hattori 2016	NR	2	5	NR	NR
Iwasaki 2016	NR	2	10	NR	NR
Kheok 2016	NR	3	10	NR	NR
Kojima 2016	NR	NR	15	NR	NR
Kumagai 2016	NR	NR	NR	NR	NR
Matsui 2016	NR	2	5	NR	NR

Agatsuma 2017	NR	"small defect"	30	NR	NR
Ahmad 2017	NR	2,5	8	NR	NR
Chou 2017	NR	NR	10	NR	NR
Hayama 2017	NR	2,5	10	NR	NR
Hirashima 2017	NR	NR	100	NR	NR
Kayano 2017	139 minutes	NR	NR	Yes	Severe
Kayano 2017	84 minutes	NR	NR	NR	NR
Kishi 2017	NR	NR	50	NR	NR
Sasaki 2017	NR	1,5	NR	NR	NR
Shibuya 2017	108 minutes	NR	NR	Yes	Mild
Nagano 2018	NR	2	6	NR	NR
Taniguchi 2018	76 minutes	NR	NR	Yes	NR

SDC 4. Table 4: Intraoperative findings

References	Nation	Number of patients	Age/sex	Clinical Signs and symptoms	History of surgery or infiammation
Benson 1964	USA	1	42/F	SBO	No
Steele 1973	USA	1	49/F	SBO	Hysterectomy
Fujioka 1976	Japan	2	61/M<br>50/M	SBO<br>SBO	No<br>No
Bircher 1981	England	2	68/F<br>68/F	SBO<br>SBO	No<br>Hysterectomy
Li 1982	China	1	69/F	SBO	No
Li 1985	China	1	62/F	SBO	Hysterectomy
Sugimoto 1985	Japan	1	38/M	SBO	No
Matsuda 1989	Japan	1	64/M	SBO	No
Mo 1991	Korea	1	59/M	SBO	No
Johnson 1992	USA	1	20/F pregnant	SBO	No
Tada 1994	Japan	1	83/M	SBO	No
Zhang 1995	China	1	51/M	SBO	No
Luo 1996	China	1	69/F	SBO, appendictis	Hysterectomy
Murakami 1996	Japan	1	80 M	SBO	No
Perez 1997	Spain	1	54/M	SBO	Cholecistectomy
Ido 1998	Japan	1	66/M	SBO	No
Igarashi 1998	Japan	1	60/M	SBO	No
Kaneko 1998	Japan	1	53/F	SBO	No
Teruya 1998	Japan	1	79/M	SBO	No
He 1999	China	1	55/M	SBO	No

Shibahara 1999	Japan	1	50/M	SBO	No
Kobayashi 2000	Japan	2	44/F<br>57/M	SBO<br>SBO	No<br>No
Endo 2001	Japan	1	69/M	SBO	No
Hokutani 2001	Japan	1	80/M	SBO	No
Sogo 2001	Japan	1	65/M	SBO	No
Takikawa 2001	Japan	1	62/M	SBO	No
Maruyama 2002	Japan	2	71/M<br>78/M	SBO<br>SBO	Gastrectomy<br>No
Sasaki 2002	Japan	1	63/M	SBO	Appendicectomy
Katayanagi 2003	Japan	1	59/M	SBO	No
Watanabe 2003	Japan	1	68/M	SBO	No
Akiyama 2004	Japan	1	39/F	SBO	Caesarean section
Koinuma 2004	Japan	1	40/F	SBO	No
Motohiro 2004	Japan	1	60/M	SBO	No
Yu 2004	China	1	81/F	SBO	No
Maruyama 2005	Japan	1	33/M	SBO	No
Mitsui 2005	Japan	1	33/F	SBO	No
Nachi 2005	Japan	1	62/M	SBO	No
Ota 2005	Japan	1	59/M	SBO	No
Shinto 2005	Japan	1	96/M	SBO	No
Van der Mieren 2005	Belgium	1	F/ Postpartum	SBO	No
Waku 2005	Japan	1	65/F	SBO	No

Yang 2005	Taiwan	1	66/M	SBO	No
Hoshikawa 2006	Japan	1	61/M	SBO	No
Nitta 2006	Japan	1	39/M	SBO	No
Isobe 2007	Japan	1	54/M	SBO	No
Zhou 2007	China	1	57/M	SBO	No
Collins 2008	Ireland	1	60/M	SBO	No
Hibino 2008	Japan	1	64/F	SBO	No
Papanikolaou 2008	Greece	1	3/F	SBO	No
Tsukahara 2008	Japan	2	34/M<br>68/M	SBO<br>SBO	No<br>No
Bao SQ 2009	China	1	62/F	SBO	No
Kan 2009	Japan	1	46/M	SBO	Appendicectomy
Murata 2009	Japan	1	80/F	SBO	No
Moriyama 2010	Japan	1	48/F	SBO	No
Nihon-yanagi 2010	Japan	1	28/M	SBO	No
Okada 2010	Japan	1	51/M	SBO	No
Oshima 2010	Japan	1	30/F	SBO	No
Takayanagi 2010	Japan	1	62/F	SBO	No
Takeshita 2010	Japan	1	56/F	SBO	No
Yang WL 2010	China	2	62/M<br>48/M	SBO<br>SBO, appendicitis	No<br>No
Agarwal 2011	India	1	78/M	SBO	No

Guo 2011	China	1	39/M	SBO	No
Harrison 2011	England	1	63/M	SBO	No
Jimmy 2011	India	1	54/F	SBO	No
Kohga 2011	Japan	1	39/M	SBO	No
Michiko 2011	Japan	2	44/M<br>45/F	SBO<br>SBO	Appendicectomy<br>No
Satomura 2011	Japan	2	79/M<br>80/M	SBO<br>SBO	No<br>No
Yazawa 2011	Japan	1	51/M	SBO	Appendicectomy
Kim 2012	Korea	1	44/F	SBO	Tubal ligation
Koshino 2012	Japan	1	81/M	SBO	No
Sawazaki 2012	Japan	1	61/M	SBO	No
Shinoto 2012	Japan	1	81/F	SBO	No
Yao 2012	China	1	60/M	SBO	No
Miura 2013	Japan	3	34/F<br>36/M<br>81/F	SBO<br>SBO<br>SBO	No<br>Laparoscopic surgery for DP<br>No
Nishida 2013	Japan	1	55/F	SBO	No
Saxena 2013	India	1	63/M	SBO	No
Toyoda 2013	Japan	2	35/F<br>37/M	SBO<br>SBO	No<br>No
Bandawar 2014	India	1	42/M	SBO	No
Li 2014	China	1	59/M	SBO	No
Fukuzawa 2014	Japan	1	39/M	SBO	No
Furukawa 2014	Japan	1	42/M	SBO	No

Hori 2014	Japan	1	46/M	SBO	No
Mandava 2014	India	1	Elderly/F	SBO	Hysterectomy
Siaperas 2014	Greece	1	28/F	SBO	Appendicectomy
Tanaka 2014	Japan	1	49/F	SBO	No
Watanabe 2014	Japan	1	26/M	SBO	No
Yang 2014	Korea	1	49/F	SBO	No
Campbell 2015	England	1	56/F	SBO	No
Farah 2015	Morocco	1	44/M	SBO	Segmental colectomy
Hirata 2015	Japan	1	35/F pregnant	SBO	No
Horii 2015	Japan	1	96/F	SBO	No
Ishita 2015	Japan	1	35/F pregnant	SBO	No
Kuroda 2015	Japan	1	64/F	SBO	No
Reddy 2015	India	1	50/M	SBO	No
Shukla 2015	India	1	62/M	SBO	No
Watanabe 2015	Japan	1	56/M	SBO	No
Benyamini 2016	Hawaii	1	40/F	SBO	Pancreatectomy, Splenectomy
Hattori 2016	Japan	1	53/M	SBO	No
Iwasaki 2016	Japan	1	50/M	SBO	No
Kheok 2016	Singapore	1	75/M	SBO	No
Kojima 2016	Japan	1	43/F	SBO	No
Kumagai 2016	Japan	1	82/M	SBO	No
Matsui 2016	Japan	1	51/M	SBO	No
Agatsuma 2017	Japan	1	17/F	SBO	No

Ahmad 2017	USA	1	55/M	SBO	No
Chou 2017	China	1	55/M	SBO	Partial descending colectomy
Hayama 2017	Japan	1	80/M	SBO	No
Hirashima 2017	Japan	1	60/M	SBO	No
Kayano 2017	Japan	2	42/F<br>53/M	SBO<br>SBO	No<br>No
Kishi 2017	Japan	1	56/M	SBO	No
Sasaki 2017	Japan	1	70/F	SBO	Appendicectomy, Ovarian tumor
Shibuya 2017	Japan	1	45/F	SBO	No
Nagano 2018	Japan	1	52/M	SBO	Appendicectomy
Taniguchi 2018	Japan	1	72/F	SBO	Sigmoidectomy

SDC 5. Type of surgical treatment

References	Time from onset to operation	Surgical procedure	Adhesiolisis	Bowel resection	Method of repair of the hernial orifice	Postoperative course
Benson 1964	7 days	Explorative laparotomy	NR	No	Interrupted sutures	Uneventful
Steele 1973	NR	Explorative laparotomy	NR	No	Interrupted sutures	Uneventful
Fujioka 1976	Day of admission<br>Day of admission	Open laparotomy<br>Open laparotomy	NR<br>NR	No<br>No	Closure the sac<br>Closure the sac	Uneventful<br>Uneventful
Bircher 1981	5 days<br>7days	Explorative laparotomy<br>Explorative laparotomy	NR<br>NR	Yes<br>No	NR<br>NR	Wound infection<br>Uneventful

Li 1982	6h	Explorative laparotomy	NR	Yes, 30 cm	NR	Uneventf ul
Li 1985	4 days	Explorative laparotomy	NR	Yes, 30 cm	NR	Uneventf ul
Sugimoto 1985	Day of admission	Open laparotomy	NR	No	NR	Uneventf ul
Matsuda 1989	Day of admission	Open laparotomy	NR	No	Interrupted slitches	Uneventf ul
Mo 1991	10 days	Explorative laparotomy	NR	Yes,40 cm	NR	Uneventf ul
Johnson 1992	7 days	Explorative laparotomy	NR	Yes, 160 cm	NR	Uneventf ul
Tada 1994	8 days	Open laparotomy	NR	No	Sutures	Uneventf ul
Zhang 1995	37 h	Explorative laparotomy	NR	Yes, 40 cm	NR	Uneventf ul
Luo 1996	24 h	Explorative laparotomy	NR	Yes, 40 cm	NR	Uneventf ul
Murakami 1996	10 days	Open laparotomy	NR	No	Sutures	Uneventf ul
Perez 1997	NR	Explorative laparotomy	NR	Yes	Sutures	Uneventf ul
Ido 1998	3 days	Open laparotomy	NR	No	Sutures	Uneventf ul
Igarashi 1998	11 days	Open laparotomy	NR	No	Interrupted sutures	Uneventf ul
Kaneko 1998	Day of admission	Emergency laparotomy	NR	Yes	NR	Uneventf ul
Teruya 1998	7 days	Open laparotomy	NR	No	Interrupted sutures	Uneventf ul
He 1999	72 h	Explorative laparotomy	NR	Yes, 100 cm	Interrupted sutures	Uneventf ul

Shibahara 1999	7 days	Open laparotomy	NR	No	Sutures	Uneventf ul
Kobayashi 2000	NR<br>21 days	Open laparotomy<br>Open laparotomy	Yes<br>NR	Yes<br>No	Sutures<br>Sutures	Uneventf ul<br>Uneventf ul
Endo 2001	NR	Open laparotomy	NR	No	NR	Uneventf ul
Okutani 2001	4 days	Open laparotomy	NR	Yes	NR	Uneventf ul
Sogo 2001	11 days	Open laparotomy	NR	No	Sutures	Uneventf ul
Takikawa 2001	9 days	Open laparotomy	NR	Yes, 7 cm	Sutures	Uneventf ul
Maruyama 2002	8h<br>22 days	Explorative laparotomy<br>Explorative laparotomy	NR<br>NR	No<br>No	Interrupted sutures<br>Interrupted sutures	Uneventf ul<br>Uneventf ul
Sasaki 2002	NR	Emergency laparoscopy and then open laparotomy	NR	Yes, 30 cm	NR	Uneventf ul
Katayanagi 2003	34 days	Open laparotomy	NR	No	Sutures	Uneventf ul
Watanabe 2003	15 days	Full laparoscopic	NR	No	NR	Uneventf ul
Akiyama 2004	14 days	Open laparotomy	NR	No	Sutures	Uneventf ul
Koinuma 2004	NR	Full laparoscopic	NR	No	Hernia stapler	Uneventf ul
Motohiro 2004	NR	Open laparotomy	NR	No	NR	Uneventf ul
Yu 2004	12 h	Explorative laparotomy	NR	Yes, 5 cm	NR	Uneventf ul

Maruyama 2005	16 days	Full laparoscopic	NR	No	NR	Uneventf ul
Mitsui 2005	10 days	Full laparoscopic	NR	No	Sutures	Uneventf ul
Nachi 2005	26 days	Open laparotomy	NR	No	Sutures	Uneventf ul
Ota 2005	16 days	Open laparotomy	NR	No	Sutures	Uneventf ul
Shinto 2005	15 days	Open laparotomy	NR	No	Sutures	Uneventf ul
Van der Mieren 2005	Day of admission	Full laparoscopic	NR	No	Sutures	Uneventf ul
Waku 2005	6 days	Open laparotomy	NR	No	Sutures	Uneventf ul
Yang 2005	48 h	Emergency laparotomy	NR	No	Sutures	Uneventf ul
Hoshikawa 2006	13 days	Open laparotomy	NR	No	Sutures	Uneventf ul
Nitta 2006	16 days	Full laparoscopic	NR	No	Sutures	Uneventf ul
Isobe 2007	6 days	Full laparoscopic	NR	No	Sutures	Uneventf ul
Zhou 2007	48 h	Explorative laparotomy	NR	Yes, 20 cm	Sutures	Uneventf ul
Collins 2008	48 h	Explorative laparotomy	NR	No	Interrupted sutures	Uneventf ul
Hibino 2008	NR	Open laparotomy	NR	Yes, 60 cm	Sutures	Uneventf ul
Papanikolao u 2008	12 h	Explorative laparotomy	NR	Yes, 60 cm	NR	Uneventf ul

Tsukahara 2008	NR<br>NR	Open laparotomy<br>Open laparotomy	NR<br>NR	Yes<br>No	Interrupted sutures<br>Interrupted sutures	Uneventful<br>Uneventful
Bao SQ 2009	37 h	Explorative laparotomy	NR	Yes, 12 cm	NR	Uneventful
Kan 2009	18 days	Explorative laparotomy	NR	Yes	NR	Uneventful
Murata 2009	13 days	Open laparotomy	NR	No	Interrupted sutures	Uneventful
Moriyama 2010	15 h	Open laparotomy	NR	No	Sutures	Uneventful
Nihon-Yanagi 2010	NR	Explorative laparotomy	Yes	No	Running sutures	Uneventful
Okada 2010	7 days	Explorative laparotomy	NR	Yes	Sutures	Uneventful
Oshima 2010	NR	Open laparotomy	NR	No	Sutures	Uneventful
Takayanagi 2010	15 days	Open laparotomy	NR	NR	Sutures	Uneventful
Takeshita 2010	7 days	Explorative laparotomy	NR	No	NR	Uneventful
Yang WL 2010	9h<br>24 h	Explorative laparotomy<br>Explorative laparotomy	NR<br>NR	Yes, 20 cm<br>Yes, 30 cm	NR<br>NR	Uneventful<br>Uneventful
Agarwal 2011	Day of admission	Explorative laparotomy	NR	No	Interrupted sutures	Uneventful
Guo 2011	9 h	Explorative laparotomy	NR	Yes, 65 cm	NR	Uneventful
Harrison 2011	NR	Explorative laparotomy	NR	Yes	NR	Uneventful

Jimmy 2011	NR	Full laparoscopic	NR	No	Intra-corporeal sutures	Uneventf ul
Kohga 2011	NR	Full laparoscopic	NR	No	Sutures	Uneventf ul
Michiko 2011	13 days<br>Day of admission	Explorative laparotomy<br>Emergency laparotomy	NR<br>NR	No<br>No	Sutures<br>Sutures	Uneventf ul<br>Uneventf ul
Satomura 2011	3 days<br>9 days	Full laparoscopic<br>Full laparoscopic	NR<br>NR	No<br>No	Sutures<br>Sutures	Uneventf ul<br>Uneventf ul
Yazawa 2011	NR	Full laparoscopic	No	No	Sutures	Uneventf ul
Kim 2012	NR	Explorative laparoscopy and the an open laparotomy	Yes	Yes	Non-absorbable sutures	Uneventf ul
Koshino 2012	14 days	Open laparotomy	NR	No	Sutures	Uneventf ul
Sawazaki 2012	18 days	First an explorative laparoscopy and the an open laparotomy	Yes	No	Sutures	Uneventf ul
Shinoto 2012	NR	Open laparotomy	NR	NR	NR	Uneventf ul
Yao 2012	7 h	Explorative laparotomy	NR	Yes, 30 cm	NR	Uneventf ul
Miura 2013	3 days<br>1 day<br>3 days	Open laparotomy<br>Open laparotomy<br>Open laparotomy	NR<br>NR<br>NR	No<br>No<br>No	Sutures<br>Sutures<br>Sutures	Uneventf ul<br>Uneventf ul<br>Uneventf ul

Nishida 2013	NR	Full laparoscopic	NR	No	Sutures	Uneventful
Saxena 2013	NR	Emergency laparotomy	NR	Yes	NR	Uneventful
Toyoda 2014	14 h<br>10 h	Full laparoscopic<br>Full laparoscopic	NR<br>NR	No<br>No	Sutures<br>Sutures	Uneventful<br>Uneventful
Bandawar 2014	NR	Explorative laparotomy	NR	Yes	NR	Uneventful
Fukuzawa 2014	10 days	Full laparoscopic	NR	No	NR	Uneventful
Furukawa 2014	13 days	Emergency laparoscopy	NR	No	Sutures	Uneventful
Hori 2014	24 h	Full laparoscopic	NR	Yes	Sutures	Uneventful
Li 2014	5 days	Emergency laparotomy	NR	No	NR	Uneventful
Mandava 2014	Day of admission	Emergency laparotomy	NR	Yes	NR	Uneventful
Siaperas 2014	NR	Diagnostic laparoscopy and then a mini-laparotomy	NR	Yes	Interrupted sutures	Uneventful
Tanaka 2014	Day of admission	Emergency laparotomy	Yes	No	NR	Uneventful
Watanabe 2014	6 days	Full laparoscopic	Yes	No	Interrupted sutures	Uneventful
Yang 2014	2 days	Full laparoscopic	NR	No	NR	Uneventful
Campbel 2015	Day of admission	Full laparoscopic	NR	No	Intra-corporeal sutures	Uneventful
Farah 2015	NR	Explorative laparotomy	NR	No	Interrupted sutures	Uneventful

Hirata 2015	3 days	Caesarian section, laparotomy	NR	No	NR	Uneventf ul
Horii 2015	Day of admission	Emergency laparotomy	NR	Yes	NR	Death for AP 46 days after surgery
Ishita 2015	3 days	Caesarian section, laparotomy	NR	NR	Sutures	Uneventf ul
Kuroda 2015	9 days	Full laparoscopic	NR	Yes, 5 cm	Intra-corporeal sutures	Uneventf ul
Reddy 2015	5 days	Explorative laparotomy	NR	No	NR	Uneventf ul
Shukla 2015	NR	Explorative laparotomy	NR	Yes	NR	Death for RD 2 months later
Watanabe 2015	3 days	Full laparoscopic	NR	No	Sutures	Uneventf ul
Benyamini 2016	48 h	Full laparoscopic	NR	No	NR	Uneventf ul
Hattori 2016	48 h	Full laparoscopic	NR	No	Non-absorbable sutures	Uneventf ul
Iwasaki 2016	7 days	Open laparotomy	NR	No	Sutures	Uneventf ul
Kheok 2016	NR	Explorative laparotomy	NR	Yes	NR	Uneventf ul
Kojima 2016	NR	Full Laparoscopic	NR	No	NR	Uneventf ul
Kumagai 2016	14 days	Full laparoscopic	NR	No	NR	Uneventf ul
Matsui 2016	4 days	Full laparoscopic	NR	No	Interrupted sutures	Uneventf ul

Agatsuma 2017	NR	Open laparotomy	NR	No	Running sutures	Uneventf ul
Ahmad 2017	24 h	Explorative laparotomy	NR	No	Absorbable sutures	Uneventf ul
Chou 2017	5 days	Full Laparoscopic	NR	No	NR	Uneventf ul
Hayama 2017	9 days	Full laparoscopic	NR	No	Opening hernia orifice	Uneventf ul
Hirashima 2017	NR	Explorative laparotomy	NR	No	NR	Uneventf ul
Kayano 2017	7 days<br>7days	Full laparoscopic<br>Full laparoscopic	Yes<br>NR	Yes<br>No	Intracorporeal sutures<br>Intracorporeal sutures	Uneventf ul<br>Uneventf ul
Kishi 2017	NR	Full laparoscopic	NR	No	NR	Uneventf ul
Sasaki 2017	NR	Full laparoscopic	NR	No	Sutures	Uneventf ul
Shibuya 2017	14 days	Full aparoscopic	Yes	No	NR	Uneventf ul
Nagano 2018	9 days	Open laparotomy	NR	No	Absorbable sutures	Uneventf ul
Tanighuchi 2018	NR	First SPS and then laparotomy	Yes	No	NR	Uneventf ul

SDC 6: Characteristics of the defect and incarcerated small bowel

References	Diameter of defect (cm)	Length of incarcerated small bowel (cm)
Benson 1964	2	15
Steele 1973	2	NR
Fujioka 1976	2,5<br>1,5	3<br>5

Bircher 1981	NR<br>NR	NR<br>NR
Li 1982	4	NR
Li 1985	2	NR
Sugimoto 1985	3	15
Matsuda 1989	3	10
Mo 1991	3	NR
Johnson 1992	5	NR
Tada 1994	2	10
Zhang 1995	3	NR
Luo 1996	3	NR
Murakami 1996	1,5	5
Perez 1997	NR	NR
Ido 1998	1	NR
Igarashi 1998	2,1	7,5
Kaneko 1998	2,5	150
Teruya 1998	1,5	10
He 1999	3	NR
Shibahara 1999	1,5	NR
Kobayashi 2000	2<br>2	20<br>10
Endo 2001	2	130
Okutani 2001	10	70
Sogo 2001	3	NR
Takikawa 2001	2	70
Maruyama 2002	3<br>2,5	5<br>5
Sasaki 2002	3,5	30
Katayanagi 2003	3	15
Watanabe 2003	NR	10
Akiyama 2004	2	4
Koinuma 2004	2	NR

Motohiro 2004	5	50
Yu 2004	5	NR
Maruyama 2005	2,5	NR
Mitsui 2005	3	15
Nachi 2005	2,5	10
Ota 2005	3	NR
Shinto 2005	2	NR
Van der Mieren 2005	NR	NR
Waku 2005	2,5	NR
Yang 2005	NR	20
Hoshikawa 2006	2	NR
Nitta 2006	2,5	NR
Isobe 2007	3	4
Zhou 2007	3	NR
Collins 2008	4	NR
Hibino 2008	3	40
Papanikolaou 2008	5	NR
Tsukahara 2008	3<br>2	10<br>20
Bao SQ 2009	3	NR
Kan 2009	4	NR
Murata 2009	2	4
Moriyama 2010	2	15
Nihon-Yanagi 2010	3	NR
Okada 2010	1,5	10
Oshima 2010	1	10
Takayanagi 2010	2	NR
Takeshita 2010	2,5	20
Yang WL 2010	5<br>4	NR<br>NR

Agarwal 2011	1,8	15
Guo 2011	4	NR
Harrison 2011	NR	NR
Jimmy 2011	2	NR
Kohga 2011	3	NR
Michiko 2011	1,5<br>2	7<br>NR
Satomura 2011	4<br>3	8<br>10
Yazawa 2011	1,5	10
Kim 2012	3	150
Koshino 2012	2	10
Sawazaki 2012	2	10
Shinoto 2012	2	150
Yao 2012	4	NR
Miura 2013	2<br>3<br>1,5	NR<br>NR<br>15
Nishida 2013	2	10
Saxena 2013	4	50
Toyoda 2013	2<br>1	20<br>75
Bandawar 2014	2	25
Fukuzawa 2014	NR	NR
Furukawa 2014	2	10
Hori 2014	3	NR
Li 2014	2,5	15
Mandava 2014	NR	NR
Siaperas 2014	3	NR
Tanaka 2014	2	5
Watanabe 2014	3	10
Yang 2014	2	15

Campbel 2015	3	6
Farah 2015	4,5	NR
Hirata 2015	2	NR
Horii 2015	2	30
Ishita 2015	2	NR
Kuroda 2015	3	10
Reddy 2015	NR	NR
Shukla 2015	2	NR
Watanabe 2015	3	15
Benyamini 2016	2	NR
Hattori 2016	2	5
Iwasaki 2016	2	10
Kheok 2016	3	10
Kojima 2016	NR	15
Kumagai 2016	NR	NR
Matsui 2016	2	5
Agatsuma 2017	“small defect”	30
Ahmad 2017	2,5	8
Chou 2017	NR	10
Hayama 2017	2,5	10
Hirashima 2017	NR	100
Kayano 2017	NR	NR
Kayano 2017	NR	NR
Kishi 2017	NR	50
Sasaki 2017	1,5	NR
Shibuya 2017	NR	NR
Nagano 2018	2	6
Taniguchi 2018	NR	NR
